# Heart blood flow simulation: a perspective review

**DOI:** 10.1186/s12938-016-0224-8

**Published:** 2016-08-25

**Authors:** Siamak N. Doost, Dhanjoo Ghista, Boyang Su, Liang Zhong, Yosry S. Morsi

**Affiliations:** 1 Biomechanics and Tissue Engineering Lab, Faculty of Science, Engineering and Technology, Swinburne University of Technology, Melbourne, Australia; 2 University 2020 Foundation, Northborough, MA USA; 3 National Heart Research Institute Singapore, National Heart Centre Singapore, 5 Hospital Drive, 169609 Singapore, Singapore; 4 Duke-NUS Medical School, Singapore, Singapore

**Keywords:** Hemodynamics, Left ventricle (LV), Computational fluid dynamics (CFD), Fluid structure interaction (FSI), Cardiovascular diseases (CVDs)

## Abstract

Cardiovascular disease (CVD), the leading cause of death today, incorporates a wide range of cardiovascular system malfunctions that affect heart functionality. It is believed that the hemodynamic loads exerted on the cardiovascular system, the left ventricle (LV) in particular, are the leading cause of CVD initiation and propagation. Moreover, it is believed that the diagnosis and prognosis of CVD at an early stage could reduce its high mortality and morbidity rate. Therefore, a set of robust clinical cardiovascular assessment tools has been introduced to compute the cardiovascular hemodynamics in order to provide useful insights to physicians to recognize indicators leading to CVD and also to aid the diagnosis of CVD. Recently, a combination of computational fluid dynamics (CFD) and different medical imaging tools, image-based CFD (IB-CFD), has been widely employed for cardiovascular functional assessment by providing reliable hemodynamic parameters. Even though the capability of CFD to provide reliable flow dynamics in general fluid mechanics problems has been widely demonstrated for many years, up to now, the clinical implications of the IB-CFD patient-specific LVs have not been applicable due to its limitations and complications. In this paper, we review investigations conducted to numerically simulate patient-specific human LV over the past 15 years using IB-CFD methods. Firstly, we divide different studies according to the different LV types (physiological and different pathological conditions) that have been chosen to reconstruct the geometry, and then discuss their contributions, methodologies, limitations, and findings. In this regard, we have studied *CFD simulations of intraventricular flows* and related cardiology insights, for (i) Physiological patient-specific LV models, (ii) Pathological heart patient-specific models, including myocardial infarction, dilated cardiomyopathy, hypertrophic cardiomyopathy and hypoplastic left heart syndrome. Finally, we discuss the current stage of the IB-CFD LV simulations in order to mimic realistic hemodynamics of patient-specific LVs. We can conclude that heart flow simulation is on the right track for developing into a useful clinical tool for heart function assessment, by (i) incorporating most of heart structures’ (such as heart valves) operations, and (ii) providing useful diagnostic indices based hemodynamic parameters, for routine adoption in clinical usage.

## Background

Cardiovascular disease (CVD) refers to abnormalities and/or the malfunction of cardiovascular components that affect the functionality of the heart. It is well known that CVD is the leading cause of mortality and morbidity in the world, particularly in developed countries. The diagnosis and prognosis of CVD in the early stage can help to reduce its high mortality and morbidity rate. Therefore, it is essential to develop various tools to enhance our knowledge of cardiovascular physiological phenomena and processes that contribute to the initiation and progression of various CVDs. The flow-induced (i.e. hemodynamic) loads are vital keys to cardiovascular structural development during the embryonic period and the formation of any change in the shape or functionality of the cardiovascular system after birth [1]. Therefore, analyzing the hemodynamic flow patterns and parameters of patient-specific heart models using various clinical tools can provide physicians with useful insights into the indicators leading to CVD, and can also assist in the diagnosis of CVD.

One clinical cardiovascular assessment tool is the catheter, an invasive medical instrument that measures blood flow or pressure. The main challenge when using traditional invasive medical tools is the occurrence of complications during and/or after operation [2]. Another robust set of clinical cardiovascular assessment tools is non-invasive medical imaging techniques, such as magnetic resonance imaging (MRI), echocardiography (ECG), and computed tomography (CT), which are able to provide valuable information on the cardiac system without the associated risks posed by traditional clinical tools. Despite the frequent use of medical imaging methods, there are some limitations and difficulties associated with heart pathology prognosis and detection in clinical practice. For instance, computed tomography (CT) is unable to provide some essential hemodynamics of blood flow patterns that can aid the early diagnosis of CVD [3]. Magnetic resonance image (MRI) images have fair spatiotemporal resolution to capture the small scale and temporal hemodynamic features of the heart. 4D MRI is a cutting edge tool to visualize the three-dimensional (3D) flow evolution over cardiac cycles by combining 3D spatial encoding and the 3D velocity-encode phase contrast method [4]. As stated in [4], the scan time is relatively long, of the order of 20 min or more, with spatial and temporal resolutions of 2–3 mm and 40–50 ms, respectively. One major drawback of 4D MRI, however, is that this technique fails to capture accurately the hemodynamic parameters, such as WSS, due to the low resolution [5], while they can be measured by computational fluid dynamics (CFD) simulation with sufficient accuracy.

 CFD has been widely used in the assessment of cardiac functionality, in combination with medical imaging techniques and even invasive medical tools. CFD is a branch of fluid mechanics that utilizes different computational techniques to analyze fluid flow behavior and patterns. CFD is capable of providing valuable hemodynamics which is useful in the clinical assessment of heart performance and the early diagnosis of heart dysfunction [3, 6, 7]. In the cardiovascular system, the left ventricle (LV) constitutes one of the most challenging domains in the application of CFD, due to its significance in the initiation and propagation of CVD, leading to heart failure (HF). It is believed that early cardiac dysfunction can be detected by analyzing the hemodynamics within the LV chamber, due to the fact that abnormal LV flow patterns are associated with reduced myocardial contractility which causes the heart to be incapable of ejecting adequate cardiac output leading to heart failure (HF) [8]. Accordingly, enormous investigations have been carried out to computationally and/or experimentally analyze the hemodynamics of the human heart and specifically of the LV.

The history of attempts to analyze LV hemodynamics dates back to 1970, when Bellhouse [9] studied blood flow dynamics in the LV. However, more recently, several investigations have been performed by the numerical simulation of intraventricular blood flow using idealized models [10, 11] or by using normal-subject LV (physiological) [2, 12] and patient-subject LV (pathological) [7, 13]. Some of the problematic challenges faced by the numerical simulation of the LV are the complexity of heart morphology, the large deformation of the heart wall during the cardiac cycle, the effect of heart valves opening and closing on the heart geometry, the electrical-fluid-structure interaction (EFSI) phenomenon involved in developing intraventricular blood flow, and finally, the transitional blood flow between the laminar and the turbulent flows during the cardiac cycle [14]. Consequently, despite the extensive investigations that have been done in this area over the last couple of decades, the numerical simulation of intraventricular blood flow in patient-specific hearts is still clinically unavailable, and needs further investigation to provide reliable and realistic results [15].

Patient-specific LV CFD simulation aims to mimic realistic cardiovascular hemodynamics to evaluate the intraventricular hemodynamics for different purposes, such as for diagnostic analysis [2], analysis of preoperative and postoperative LVs to evaluate surgical outcomes [13], preoperative LV analysis to examine various surgical alternatives to choose the best option [16], and finally, the analysis of pathological LVs to assess their physiological conditions [17]. Table 1 summarizes the works published over the past 15 years on the simulation of human patient-specific LVs. The purpose of this review paper is to comprehensively discuss and explain recent CFD investigations of human patient-specific LVs. In this review paper, we discuss the different CFD methodologies employed to simulate intraventricular flows as well as elucidate the numerical investigations and findings of the published works. Moreover, the clinical implications of this research are also discussed in our paper. Finally, we discuss CFD shortcomings and the future direction of CFD simulations of patient-specific LVs.

**Table 1:** Summary of the published papers that simulate patient-specific LVs

First author	Year	Geometry				Imaging technique	CFD method^b^	Phase^c^	Validation results^d^	CFD Solver
		Condition^a^	Type	Number of case studies						
				Normal	Patient					
Doost [18]	2016	Normal	3D	1	–	MRI	GP	FCC	N/A	ANSYS-fluent
Su [19]	2016	Normal and PAH	3D	1	1	MRI	GP	FCC	N/A	ANSYS-fluent
Nguyen[20]	2015	Normal person and patients with diastolic dysfunction	3D	4	2	MRI	GP	FCC	Blood flow pattern is qualitatively validated using echocardiography PIV	In-house CFD solver
Muehlhausen [21]	2015	Normal	3D	1	–	MRI	GP	FCC	N/A	Coupled fluent, MpCCI, and Abaqus
Su [22]	2014	HCM	3D	1	1	MRI	GP	FCC	N/A	ANSYS-fluent 14
Su [23]	2014	Normal	2D	1	–	MRI	GP	FCC	N/A	ANSYS-fluent 14
Khalafvand [13]	2014	Before and after SVR and normal	3D	1	2	MRI	GP	FCC	N/A	ANSYS-fluent 12
Moosavi [2]	2014	Normal	3D	1	–	MRI	GP	FCC	N/A	ADINA v8.6
Seo [12]	2014	Normal	3D	1	–	CT scan	GP	FCC	N/A	N/A
Chnafa [14]	2014	Not specified	3D	1	–	MRI	GP,LES	FCC	N/A	YALES2BIO
Corsini [16]	2014	SV	3D	1	–	MRI	Multi-scale	FCC	The flow obtained from the multi-scale analysis is validated using the clinical MR and echo-Doppler data	Simnon
Vecchi [24]	2014	Normal and HLHS	3D	1	1	ECG	GP	FP	N/A	CHeart
Seo [25]	2013	Normal	3D	1	–	CT scan	GP	FCC	N/A	N/A
Mangual [7]	2013	DCM and Normal	3D	20	8	ECG	IBM	FCC	N/A	N/A
Vecchi [26]	2013	HLHS	3D	1	2	MRI	GP	FP	Myocardial shape parameters is validated using dual phase MRI	N/A
Nguyen [27]	2013	Normal	3D	1	–	MRI	GP	FCC	N/A	N/A
Le [28]	2013	Normal	3D	1	–	MRI	IBM	FP	N/A	FSI-CURVIB flow solver
Dahl [29]	2012	Normal	2D	1	–	Ultrasound	GP	FP	N/A	ANSYS-fluent 6.3.26
Lassila [30]	2012	Mild mitral regurgitation, MI and stroke	3D	–	1	MRI	GP, Multi-scale	FCC	The flow rate and pressure obtained from the multi-scale analysis are validated using a closed loop hydraulic system	Open-source LifeV code
Khalafvand [17]	2012	MI	2D	3	3	MRI	GP	FCC	N/A	ANSYS-CFX12
Khalafvand [31]	2012	Before and after SVR	3D	–	1	MRI	GP	FCC	N/A	ANSYS-CFX12
Le [32]	2012	Normal	3D	–	1	MRI	IBM	FCC	N/A	FSI-CURVIB flow solver
Mihalef [33]	2011	Normal	3D	1	–	CT scan	GP	FCC	N/A	N/A
Krittian [34]	2010	Normal	3D	1	–	MRI	GP, coupled-FSI	FCC	The flow pattern predicted by CFD simulation is qualitatively validated using a circulatory system	Coupled fluent, MpCCI, and Abaqus
Doenst [35]	2009	Before and after SVR	3D	–	1	MRI	GP	FCC	The flow pattern predicted by CFD simulation is qualitatively validated using a circulatory system	Coupled Fluent, MpCCI, and Abaqus
Schenkel [36]	2009	Normal	3D	1	–	MRI	GP	FCC	The flow pattern predicted by CFD simulation is qualitatively validated using MRI velocity mapping	Star-CD
Long [8]	2007	Normal	3D	6	–	MRI	GP	FCC	The flow pattern predicted by CFD simulation is qualitatively validated using MRI velocity mapping	ANSYS-CFX4
Liang [37]	2007	LHF and normal	3D	1	1	N/A	GP, Multi-scale	FCC	The flow obtained from multi-scale analysis is validated using echo-Doppler data	In-house C ++ code
Long [38]	2003	Normal	3D	1	–	MRI	GP	FCC	N/A	ANSYS-CFX4
Saber [39]	2001	Normal	3D	1	–	MRI	GP	FCC	N/A	STAR–CD
Saber [40]	2001	Normal	3D	1	–	MRI	GP	FCC	N/A	STAR–CD

^a^Condition (*DCM* dilated cardiomyopathy, *HCM* hypertrophic cardiomyopathy, *HLHS* hypoplastic left heart syndrome, *LHD* left heart failure, *MI* myocardial infarction, *PAH* pulmonary arterial hypertension, *SV* single ventricle, *SVR* surgical ventricular reconstruction)

^b^Solution method (*GP* geometry-prescribed, *FSI* fluid-structure interaction, *IBM* immersed boundary method, *LES* large eddy simulation)

^c^Phase (*FCC* full cardiac cycle, filling phase)

^d^Validation (*PIV* particle image velocimetry)

## Computational fluid dynamics (CFD) Approaches

**Fig. 1:**
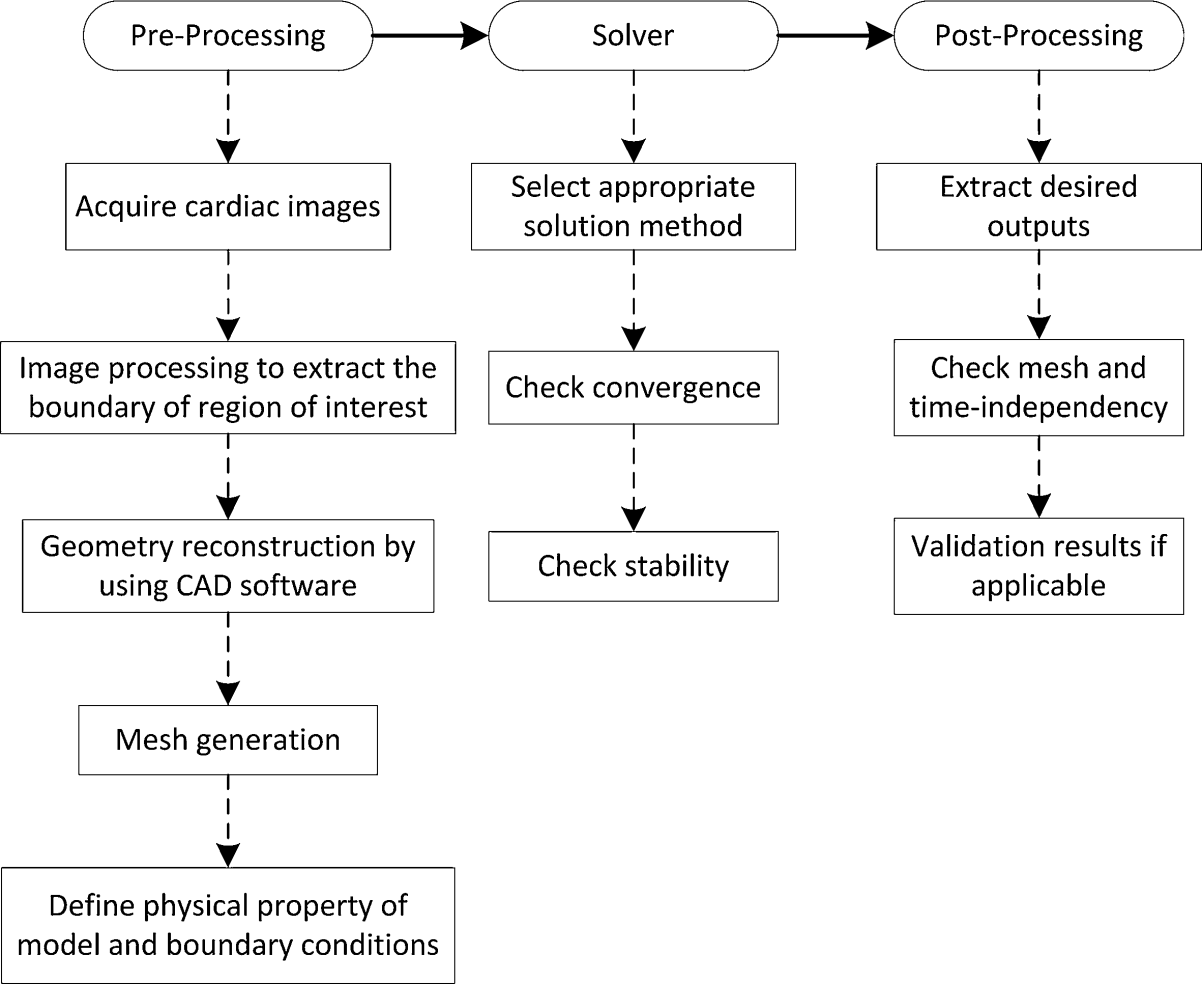
Main stages required to perform IB-CFD simulation in general

Generally, each CFD simulation has three main components: a pre-processor, solver, and post-processor. In the IB-CFD method, generally, each step consists of different substeps, as illustrated in Fig. 1. The details of the substeps depend on the numerical approach chosen to perform the simulation of the patient-specific LV. Typically, there are two main approaches for the numerical simulation of LV using CFD techniques: (i) the geometry-prescribed method that solves only the fluid domain by prescribing the movement of the LV myocardial wall as the fluid domain boundary condition; (ii) the fluid-structure interaction (FSI) method that numerically solves the governing equations of both the fluid and structure domains by coupling the CFD and structural solver. The FSI method is further subdivided into two different approaches: fictitious FSI [32] and realistic FSI [34].

The geometry-prescribed method is based on the assumption that the flow-induced load on the LV wall is negligible in comparison to the structural-induced load on the fluid flow [36]. In this approach, the LV myocardium motion is prescribed to the numerical solver by using two different approaches: directly by extracting wall motion data from medical images [13], and indirectly by setting up some mathematical equations to formulate wall motion [41]. The latter method can be used in idealized models, but is not applicable to patient-specific models. To date, the geometry-prescribed method using medical images to define wall motion is the most popular approach to simulate LV hemodynamics due to its convenience and the available computing resources. The fictitious FSI method or the immersed boundary method (IBM) is primarily appropriate to simulate flow in heart valves, although in some of the published literature [6, 32] this method has also been successfully employed in LV CFD simulation. In this method, because the wall is not fitted to the coordinate curve, the boundary layer information is not accurate enough for use in clinical decision making. The realistic FSI method, on the other hand, couples both the CFD and structural solver (mostly the finite element solver), to simulate both the fluid and structure domains simultaneously. This method is hence more complicated and also more numerically expensive (both time-consuming and requiring more sophisticated computing recourse) for the CFD modeling of the intra-LV blood flow.

The Lagrangian and Eulerian are the two methodologies that describe material kinematics. In the Lagrangian approach, the observer tracks the individual particles of the material as they move through space and time. In the Eulerian approach, the observer stands at a fixed point, and the kinematic quantities of the physical properties of the material at the fixed point are described as functions of time, as the time is passing regardless of the specific particles of the material; in the Eulerian method, the continuum mechanics framework is used to formulate the material kinematics. However, the Lagrangian and Eulerian methods are mainly used to numerically simulate the kinematics of fluid and solid materials, respectively. To numerically simulate FSI-applied problems (such as to numerically simulate intraventricular flow), neither the Eulerian nor the Lagrangian formulation is applicable to simulate the structure and fluid domains [42, 43]. To formulate the governing equations of the fluid and structure domains, an arbitrary description of the boundary is required to follow the motion of the boundary, with the mesh motion neither spatially fixed similar to the Eulerian method nor attached to the material to follow the boundary particles similar to the Lagrangian method [44].

The new technique to describe material kinematics is called the arbitrary Lagrangian–Eulerian (ALE) description, which is considered to be one of the most effective ways to analyze FSI problems involving both small and large structural deformations. In this approach, the flow domain is time-dependent, and the interface boundaries can be changed as the structure deforms [42]. In both the geometry-prescribed and the FSI approaches, the ALE approach is used for the formulation of the governing equations. The integral forms of continuity and momentum equations (Navier–Stokes equation) of the fluid domain are written as [23]:

1 $$\frac{\partial }{{\partial t}}\int_V {\rho dV} + \int_S {\rho \left( {\vec v - \overrightarrow {{v_b}} } \right) \cdot \vec ndS} = 0$$

2 $$\frac{\partial }{{\partial t}}\int_V {\rho \vec vdV} + \int_S {\left( {\rho \vec v\left( {\vec v - \overrightarrow {{v_b}} } \right) + pI - \vec \tau } \right) \cdot \vec ndS} = 0$$

where *ρ* is the fluid density; $\vec{v}$ is the velocity vector of fluid; $\overrightarrow {{v_{b} }}$ is the velocity vector of the moving boundary; $\vec{n}$ is the outwardly directed vector normal to *dS*; *S* is the boundary of the control volume, *V*; *p* is the pressure; *I* is the unit tensor; and $\vec{\tau}$ is the viscous stress tensor. The blood viscosity has been mostly assumed to be constant (*ρ* = 1050 kg/m^3^) in all published papers, owing to blood incompressibility. Moreover, blood viscosity has been assumed to be constant in most published papers by using the dynamic viscosity of *μ* = 0.0035 Pa.s, but in some papers blood has been assumed to be a non-Newtonian fluid by utilizing the Carreau–Yasuda model [36] and the Carreau [34] model. In many publications [45–51], it has been shown that blood significantly possesses the non-Newtonian properties, such as shear thinning, viscoelasticity, and thixotropic. In our most recent publications [18, 52], the effect of the non-Newtown assumption on the flow dynamics was analyzed by using different blood rheological models under the physiological condition. In this publication, it was demonstrated that the non-Newtonian assumption has quite a significant importance to the intraventricular hemodynamics, such as the wall shear stress (WSS). Therefore, the accuracy of the numerical analysis of the blood flow dynamics can be affected by neglecting the non-Newtonian property of the blood.

## Geometry reconstruction methods

The physiological/pathological patient-specific LV geometry must be reconstructed in order to analyze the complex intraventricular blood flow. In so doing, medical images of the patient’s heart need to be captured during a cardiac cycle and used to reconstruct the geometry by employing different image segmentation and image processing techniques. For carrying out intra-LV blood flow modeling, we are employing non-invasive medical images to reconstruct the anatomical heart models in order to use them in CFD simulation, which is called imaged-based CFD (IB-CFD) simulation. In this method, however, the LV geometry quality strongly depends on the medical imaging techniques, the spatiotemporal resolution of the obtained medical images, and the segmentation and image processing technique employed to reconstruct the geometry.

Moreover, due to the insufficient time resolution of the extracted medical images during one cardiac cycle to employ in the numerical simulation, extra intermediate images between the main images must be produced by using an appropriate interpolation method. In several papers, such as [36], this interpolation approach for obtaining more information has been thoroughly explained. The number of intermediate images must be such that the courant number be close to one for the convergence/stability criteria of numerical simulation [31]. However, generally patient-specific geometry reconstruction is cumbersome and time consuming. The IB-CFD simulation needs various operator-dependent steps that include image acquisition, image segmentation, geometry reconstruction, mesh generation, and finally numerical simulation [27]. The operator-dependent steps of IB-CFD could probably be sources of error that can impact on the result accuracy [27].

## Boundary conditions

### Different types of boundary conditions

In order to conduct numerical simulation, a proper set of boundary conditions should be imposed on all boundaries. The numerical results significantly depend on the type and the accuracy of the boundary conditions. Therefore, any incorrect boundary conditions will lead to the reproduction of incorrect results which can affect a clinical decision based on the numerical results. In the numerical simulation of the LV, the geometry is mainly divided into two parts with different types of boundary conditions:

#### Myocardial wall

The moving wall and no-slip boundary conditions are required to be imposed on the myocardial wall with different strategies, depending on the simulation approach. In coupled FSI [21], the myocardial wall motion should be set to be automatically derived from coupling the structural and CFD solvers. In the geometry-prescribed [13] and immersed-boundary [53] methods, the myocardial wall motion should be prescribed to the CFD solver. In this case, the spatiotemporal node positions should be derived after geometry reconstruction in order to import into the CFD solver.

#### Mitral and aortic annulus

The combination of inflow or outflow with the wall boundary conditions needs to be imposed on the mitral and aortic annulus, regardless of the presence or absence of the valve leaflets in the simulation. The wall boundary conditions (i.e., completely closed) should be defined in the mitral and aortic orifices during systole and diastole, respectively. Additionally, the inflow and outflow boundary conditions should be selected in the mitral and aortic orifices during diastole and systole, respectively. For the inflow or outflow period of the cycle, time-variant pressure [34], velocity [39], or flux [53] should be imposed on the mitral/aortic orifices. However, different types of mitral/aortic orifices have been implemented in the literature, such as an orifice with a simple boundary condition [40], a hybrid orifice with a combination of a pressure and velocity profile [38], and an orifice with a different opening area over the cycle [34, 36]. The hybrid boundary condition could be an effective approach to overcome the mass conservation equation unbalance during the numerical solution involving using velocity as the inlet and outlet boundary conditions. In the case of using velocity/flux as inflow or outflow [14], because blood is an incompressible fluid, the time-variant velocity/flux profile can be obtained from the temporal variation of the LV volume (or the surface area in 2D simulation). In [25], an expression has been derived for the blood flux through the aortic and mitral orifices by dividing the cardiac cycle into five distinct phases: E-wave, diastasis, A-wave, iso-volumetric contraction, and systole. The pressure waveform boundary condition could be also be assumed to be constant [40] or a time-varying waveform [2], for using the multi-scale analysis of the entire cardiovascular system [37] or a simplified model such as the 3-element Windkessel model [21]. However, as mentioned in [40], varying the magnitude of pressure in the boundary condition will not affect the intraventricular flow dynamic due to the nature of the Navier–Stokes equations; hence, constant pressure can be used if the acquisition of intraventricular pressure is not the desirable output.

Despite many investigations having been conducted by using different types of boundary conditions, it remains unclear as to which type is more appropriate in order to more accurately simulate the LV flow dynamics [27]. Long et al. [38] used different types of boundary conditions in the inlet and outlet orifices in order to evaluate the impact of choosing different boundary conditions for the intraventricular flow dynamics, by utilizing: (i) the pressure boundary condition, (ii) the hybrid boundary condition, or a combination of the imposed pressure and velocity at valve opening, (iii) different pressure patch locations, and (iv) different orifice opening sizes. The velocity at the valves in the hybrid case was assumed to be uniform during the valve opening phase. Moreover, zero pressure was imposed on the pressure patch area in the hybrid boundary condition. However, it has been demonstrated that the intra-ventricular flow highly depends on the boundary condition. In this regard, Lassila et al. [30] examined the influence of the boundary conditions on the intraventricular flow pattern by using a combination of multi-scale and IB-CFD. In their research, they used a different boundary condition in the valve orifice. The ideal diode is used to model the valve in the multi-scale method, which allows blood flow through the valve during the positive pressure difference and prevents flow in the reverse direction during the negative pressure difference.

### Incorporating the valve leaflets

In only a few publications [12, 14, 19, 23, 28, 29, 54], valve leaflet motions have been incorporated into the patient-specific LVs. In most publications, valves have been simply modeled as fully open or fully closed orifices. However, neglecting the valve leaflet motion can affect the accuracy of the results, which may thereby influence clinical decision making based on the CFD approach. Neglecting the valve leaflet is due to the low spatiotemporal resolution of the medical images and the high-speed opening and closing of the leaflets [40]. In some researches, valves have been simulated by utilizing the rigid leaflets in both the mitral and aortic valves [19, 23], or only in mitral valves [12, 29, 54] or only in aortic valves [28]. Moreover, in [14], the valve leaflets have been reconstructed in another way by extracting the valve annulus from the visual inspection of medical images.

Two different approaches have been implemented in order to derive the motion of valve leaflets: (i) prescribing the leaflet motion to the CFD solver, and (ii) predicting the valve leaflet motion by using the FSI approach. In the first approach, the physiological leaflet kinematics should be extracted over the cardiac cycle by using images such as echocardiographic data and then prescribed to the CFD solver [12, 55]. In the second approach, the partitioned or monolithic methods can be implemented to predict leaflet motion automatically [54]. In the partitioned method, the moment equation of the leaflets and the Navier–Stokes equations are solved simultaneously to obtain the angular position of each leaflet and the moment exerted onto the leaflet interface. Then, these two equations are coupled into each other to iteratively update the moment obtained in both equations until convergence is achieved. In the monolithic method, the total moment exerted from the blood to the leaflet surface is calculated for each time-step. Subsequently, the angular acceleration and the leaflet positions are computed by substituting the exerted moment in the moment equation, without coupling to each other or the iteration. The monolithic method has not been used so far to simulate the valve leaflet motion incorporated with the LV, as it is unable to accurately predict leaflet motion in comparison to the partitioned method. Therefore, the partitioned method will be discussed briefly in the following paragraphs.

The general form of the leaflet moment equation which should be solved separately in each leaflet to predict the angular position has the following form [23]:

3 $$\ddot{\theta } + \zeta \theta = \frac{M}{I}$$

where *θ* refers to the leaflet angular position, *ζ* damping coefficient, *I* moment of inertia, and *M* the moment. The damping coefficient has been neglected in all the aforementioned papers owing to the fact that the friction force is negligible in comparison to the force exerted by the blood flow to the leaflet interface. The moment of inertia also depends on the leaflet length and thickness. However, Eq. (3) is an ordinary differential equation which can be numerically solved by using different numerical approaches, such as the first order Euler implicit discretization in [23]. The leaflet moment obtained from this equation ($I\ddot{\theta }$) and the CFD simulation (*M*_*CFD*_) should be compared to each other in order to check the convergence criteria ($\varepsilon = \left| {M_{{CFD}} - I\ddot{\theta }} \right|$). The iteration will stop once it meets the convergence criteria; otherwise, the angular position of the leaflet should be updated and the abovementioned cycle should be performed again until the convergence criterion is met.

**Fig. 2:**
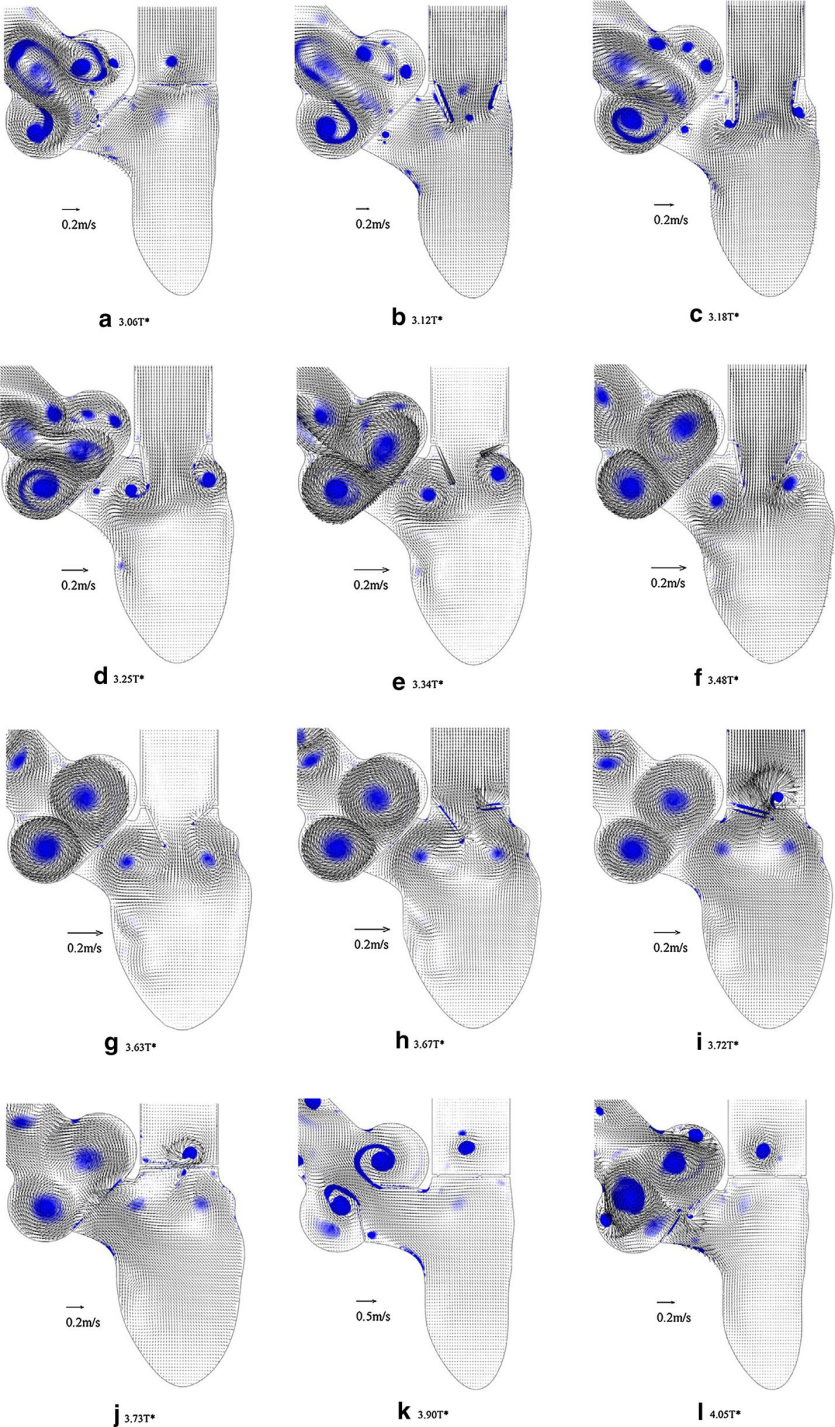
Effect of valves opening and closing on the intra-ventricular flow pattern: Both mitral and aortic valve leaflets are simulated using the rigid leaflets during the entire cardiac cycle. Despite the vortices in LA and AO, the flow field in LV is relatively uniform at the onset of diastole (**a**). Two vortices are formed in the vicinity of the mitral valve leaflet once diastole starts (**b**). As mitral valves open more, the boundary layer separation on the tip of both mitral leaflets generates two vortices (**c**, **d**). Similarly, two large vortices are formed inside the aorta after boundary separation on the tip of both aortic leaflets (**e**–**g**). The vortices are rolled up inside the LV and dissipated at the end of diastole (**h**–**j**). During aortic valve openings, a similar boundary separation is formed on the tip of leaflets (**k**). Finally, the vortices get separated and rolled up to the aorta during the aortic valve closure (**l**) [23] (Reprinted from [23], with permission from Elsevier)

A similar framework was developed by Dahl et al. [29] to integrate the motion of only the mitral valve leaflets during the diastolic phase in 2D simulation. They used ultrasound imaging to extract the angular positions of both leaflets during the filling phase in order to validate the results obtained from FSI. Their results show that both the anterior leaflet opening dynamics (with low angular velocity) and the posterior leaflet opening dynamics (with high angular velocity) are consistent with the in vivo ultrasound measurements. This framework was completed in [23] by incorporating both aortic and mitral valve leaflet motions in the entire cardiac cycle. As shown in Fig. 2, this work [23] illustrated the initiation and propagation of vortex contours within the LV and the aorta region during the cardiac cycle. The numerical results show that the opening angle of both the mitral and aortic valve leaflets is not similar during the cardiac cycle due to the asymmetric intraventricular flow pattern and non-uniform upstream flow, respectively. The mitral valve leaflet starts opening rapidly in early diastole, but is partially closed in mid-diastole and then reopens during the late diastole as the left atrium (LA) contracts. On the other hand, the aortic valve leaflet opens rapidly with the onset of systole and then closes slowly until the end of systole.

**Fig. 3:**
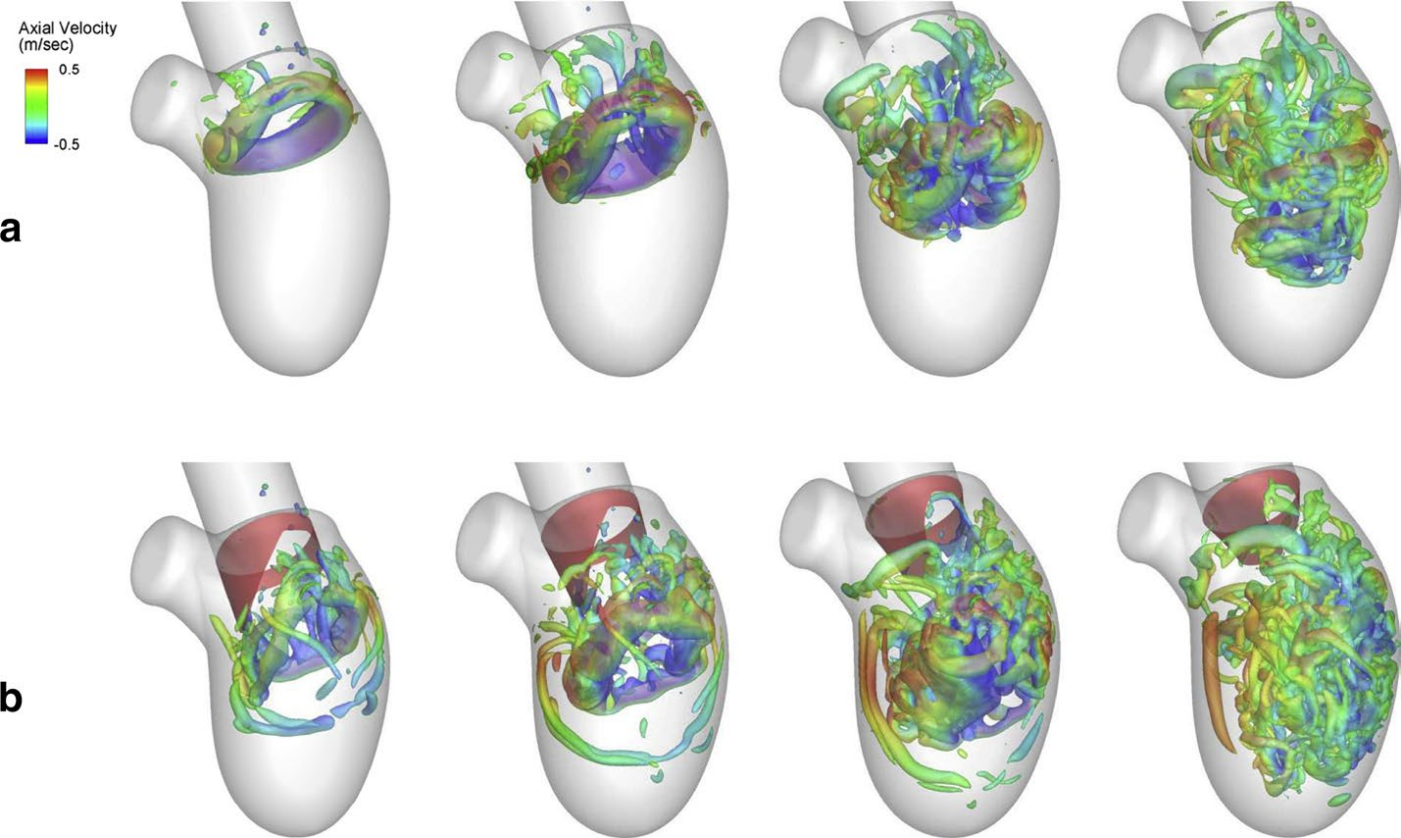
Comparison of the development of intraventricular flow with and without incorporating valve leaflets: The intraventricular vortex structure formation during the early filling phase is compared in two different conditions: **a** without the mitral valve, **b** physiological leaflet. **a** The circular major vortex ring starts to form during early diastole in the mitral annulus (t = 0.1). The vortex ring then is pinched off to the middle of LV during mid-diastole (t = 0.15 and 0.2). The major vortex rings start breaking down and propagate towards the middle of the LV at the end of diastole. The distorted vortex then penetrates up to two-thirds of the LV (t = 0.25). **b** The vortex starts breaking even in the early stage of diastole and reaches to the middle of LV (t = 0.1). As time passes, the major vortex ring propagates deeply toward the middle of LV (t = 0.15) and then starts disintegrating (t = 0.2). The distorted vortex reaches close to the LV apex at the end of diastole (t = 0.25) [12] (Reprinted from [12], with permission from AIP Publishing)

**Fig. 4:**
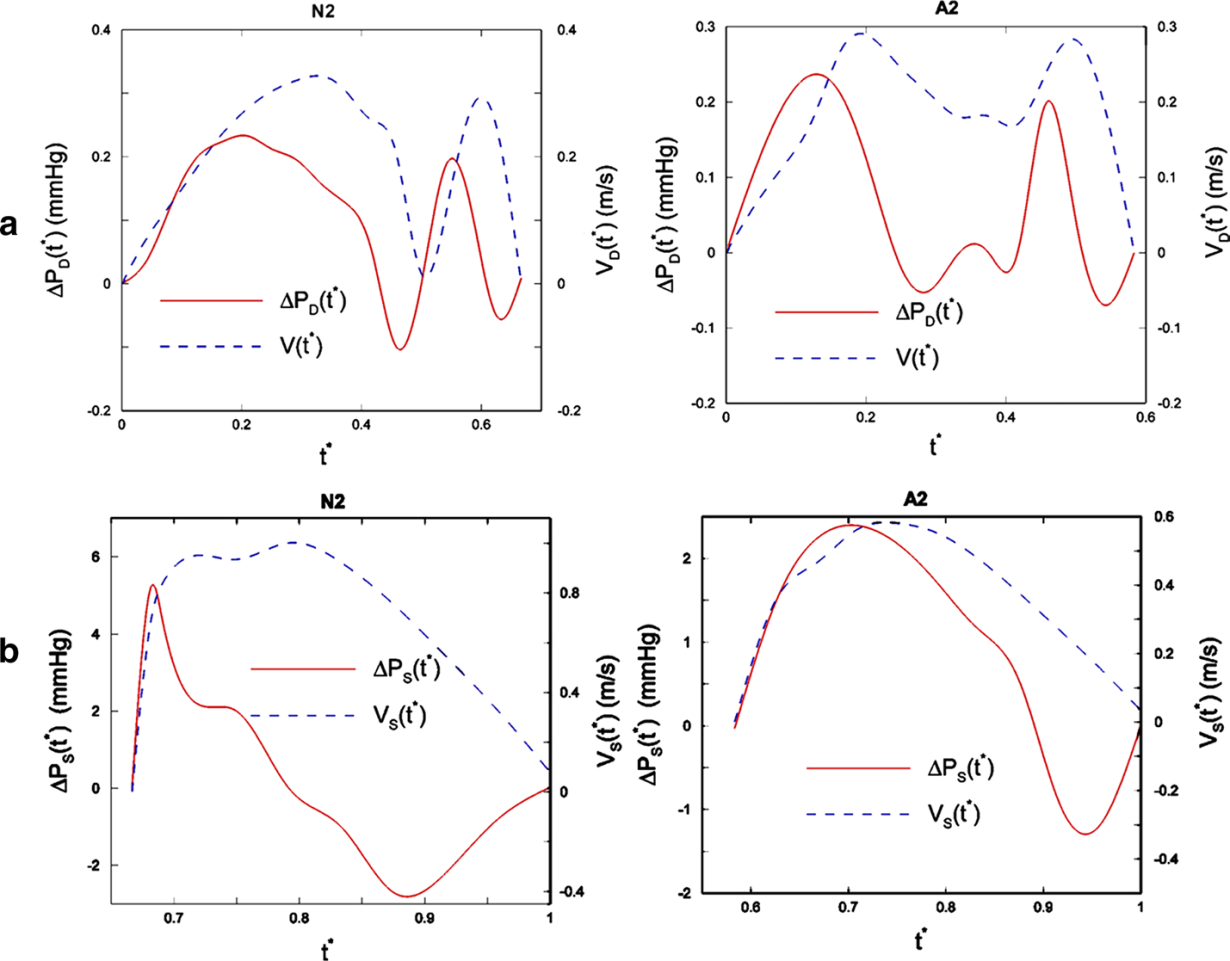
Comparison of the pressure drop in a normal subject and MI patient. **a** The velocity magnitude at the mitral and the pressure drop during diastole. **b** The velocity magnitude at the aortic orifice and the pressure drop during systole in one normal (N2) and one MI patient (A2). The pressure drop is defined as the difference in the pressure between the apex pressure and mitral orifice (during diastole) or aortic orifice (during systole) pressure. The maximum pressure occurs after A-wave and E-wave during diastole and peak of ejection during systole due to flow acceleration and deceleration [17] (Reprinted from [17], with permission from Elsevier)

In order to investigate the effect of integrating valve leaflet motion into blood flow dynamics, Seo et al. [12] integrated the mitral valve leaflets into the LV geometry and compared the results for the case without valves. As shown in Fig. 3, the incorporation of the mitral valve leaflet helps to develop the circulatory and asymmetry vortex rings during diastole. Figure 4 illustrates how the blood penetrates deeply toward the LV apex in the model including the mitral valves, in comparison with the model without the mitral leaflets. Bileaflet mechanical heart valve (BMHV) has also been incorporated into the LV in [28]. The main drawback of this research is that the authors ignored the mitral valve motion, which is more important to the intraventricular flow pattern in comparison to the aortic valve leaflet motion, because the key vortices are initiated during the diastolic phase. The evidence from this study suggests that implanting a prosthetic heart valve leads to a more complex flow pattern and causes turbulent flow inside the LV cavity which could enhance clinical complications after BMHV implantation [28]. In this investigation, the numerical results show the valve opening kinematics to be mostly symmetrical, while the closing kinematics is highly asymmetrical.

## Patient-specific study subjects

### Physiological patient-specific LV models

The physiological patient-specific LV is the subject of most published papers. In these publications, the geometry has been reconstructed by using medical images of the physiological heart in order to investigate the development of the intraventricular blood flow pattern and different hemodynamic parameters. In 2001, Saber et al. [40] proposed a methodology for IB-CFD simulation of the patient-specific human heart, and showed that this approach is able to capture the intraventricular hemodynamic parameters, such as the blood flow pattern as well as the formation and propagation of vortices during the cardiac cycle. Even though their methodology had some shortcomings by assuming a simplified LV chamber geometry, their methodology was a significant step in the simulation of the human patient-specific LV based on the IB-CFD approach. Later, they [39] improved their previous simplistic LV geometry by adding the proximal LA and ascending aorta to the geometry, improving the MRI data acquisition technique, and employing an improved interactive segmentation technique to obtain more realistic time-varying LV geometry. It should be mentioned that a small part of the aorta and LA needs to be added into the LV in order to minimize the possible inaccuracy associated with the boundary condition assumption in the aortic and mitral orifices.

Analyzing the development of intraventricular blood flow patterns or vortex propagation can produce beneficial results for use in the clinical assessment of the cardiovascular function. The qualitative and quantitative analysis of the intraventricular flow pattern by using different LV models not involving any disease is quite similar, with only a few discrepancies over the cardiac cycle. Another significant issue in the LV simulation is determining how many cycles need to be simulated to perform the post-processing step. The results in some early cycles of the simulation are unreliable owing to the inaccuracy of initial condition assumptions. Even though it was discussed in [14] that the flow is highly variable from cycle to cycle due to the intraventricular turbulent flow, it is well accepted in most publications that the flow is repeatable after a few cycles. Also, it has been shown that the flow pattern is repeated with only a small variation after the third cycle [18]. However, small variations in the flow pattern or other hemodynamic parameters can be expected in the subsequent cycles.

Ventricular blood mixing refers to the mixing of fresh blood in each cycle with the residue of blood from previous cycles [56]. In the literature, ventricular blood mixing has been found to be highly dependent on intraventricular blood dynamics [25]. Intraventricular blood mixing is an important key in providing valuable information for clinical practice to evaluate cardiac pumping performance [25]. Blood mixing also provides further information by which to evaluate the ventricular washout, which indicates the fraction of residual ventricular blood present after each cardiac cycle. A ventricle with a low washout [57] and apical stagnant flow [58] is prone to a high risk of thrombosis formation. For this purpose, Lagrangian particle tracking can be used to determine intraventricular blood mixing. Therefore, this index is significant in the clinical assessment of heart functionality utilizing the IB-CFD technique. For example, it has been shown that incorporating valve leaflet motion in the simulation can lead to better blood mixing and apical washout [12].

### Pathological heart patient-specific models

Early cardiac pumping dysfunction can be detected by analyzing LV intraventricular hemodynamics during the diastolic phase [59]. The CVD survival rate due to LV diastolic dysfunction and subsequently HF can be enhanced by early diagnosis [27]. The results of a large volume of published literature indicate that IB-CFD is potentially a promising non-invasive tool for the early diagnosis of LV dysfunction. However, the main challenging issue of IB-CFD in the prognosis of heart dysfunction is finding the correlation between the hemodynamic parameters and the risk factors that initiate heart dysfunction. For instance, it is believed that the formation of the mitral vortex ring during the filling phase is linked with different diastolic dysfunctions [32]; therefore, studying the formation and propagation of the mitral vortex ring could assist physicians in the early diagnosis of CVDs. In this section, we briefly present the different heart dysfunctions that have been simulated in pathological patient-specific LVs, and then discuss their numerical findings. However, it must be noted that up to now, there is a limited number of published papers that have attempted to simulate human patient-specific hearts, especially with pathological conditions.

#### Myocardial infarction (MI): ventricular remodeling and surgical restoration

Coronary atherosclerosis causes MI proceeding to decreased ventricular contractility, progressive heart remodeling and heart attack, which can lead to HF and sudden cardiac death. However, even for survivors of MI [8], the heart’s natural functionality continues to deteriorate during the progressive ventricular remodeling process. Therefore, analyzing the MI heart functionality and the alteration of the hemodynamic parameters during the remodeling process (to a more spherical heart shape due to reduced cardiac contractility) could assist physicians in understanding the consequences of MI. Moreover, in some cases, surgical ventricular reconstruction (SVR) is performed to treat the heart remodeling caused by MI [60, 61]. The purpose of SVR is to repair the heart functionality by reducing the enlarged heart volume and restoring the heart’s normal ellipsoidal shape (from its more spherical remodeling shape). The preoperative CFD simulation of the patient-specific heart can assist clinicians to achieve the desired outcome by analyzing the intraventricular flows in different heart shapes and sizes prior to SVR surgery in order to determine the optimal SVR procedure to obtain optimum intraventricular hemodynamics leading to improved cardiac output. Then, the postoperative CFD simulation of the patient-specific heart can also be utilized to investigate the SVR outcome [61, 62].

**Fig. 5:**
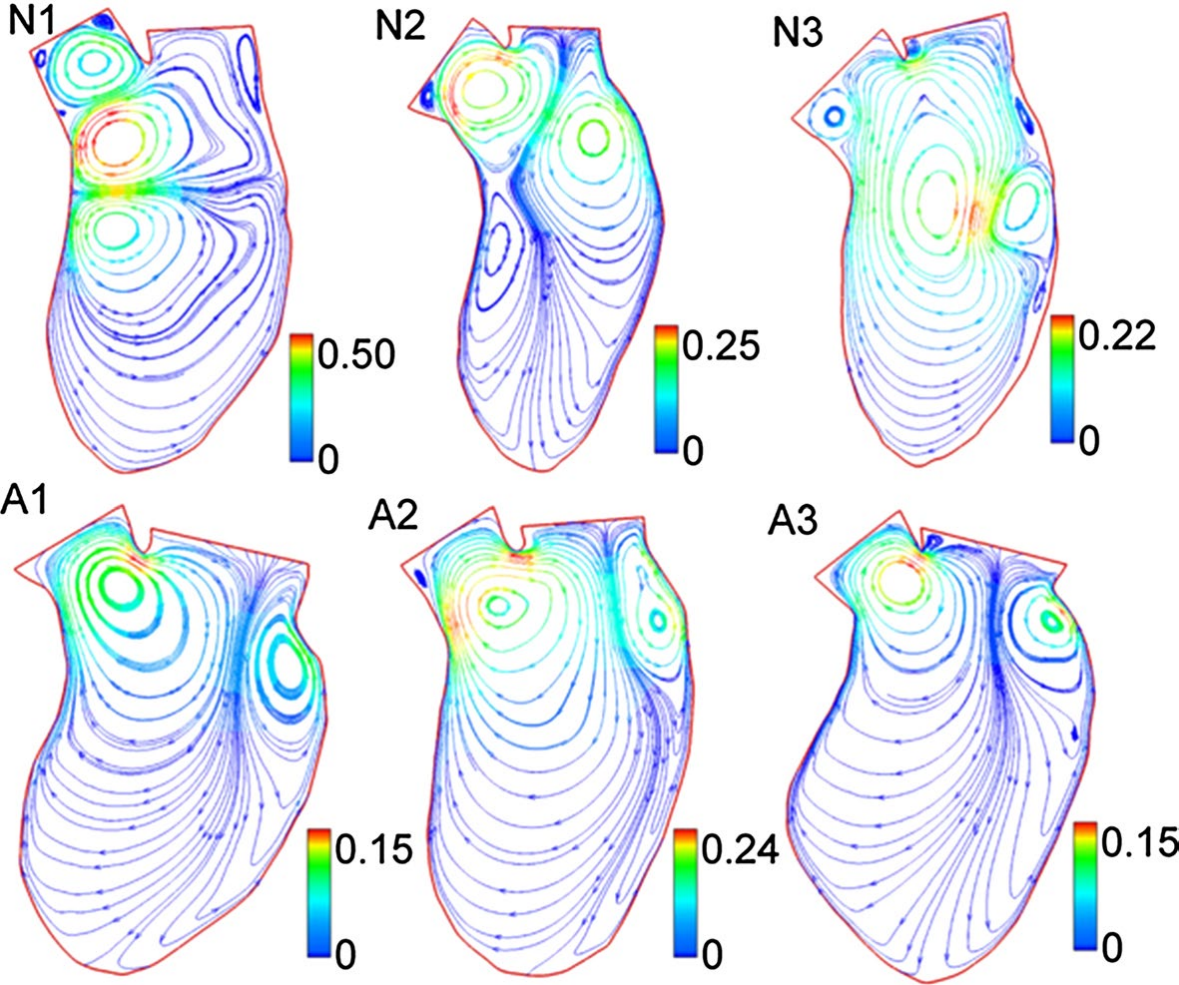
Comparison of intraventricular flow patterns in a normal subject and MI patient. The figure illustrates the streamlines at the end of diastole for normal cases (N1, N2, N3) and abnormal cases (A1, A2, A3). It can be noted that more vortices are generated in the normal LVs. It is seen that the inside of the LV cavity is dominated by a big vortex in the N3 case and all other abnormal cases [17]. ​(Reprinted from [17], with permission from Elsevier)

Even though the 3D model of the LV can reveal more realistic cardiovascular hemodynamic characteristics, it is accepted that 2D modeling is also quite capable of capturing the main hemodynamic characteristics during the cardiac cycle. In this regard, Khalafvand et al. [17] studied three different normal LVs and three different patient LVs after MI, to investigate the effect of heart remodeling on the hemodynamic parameters. In this simulation, they thoroughly demonstrated the formation and propagation of vortices, and compared the flow patterns of all cases during the entire cardiac cycle. Also, as shown in Fig. 4, they plotted the pressure difference (between the mitral and aortic orifices and the LV apex) in the LV for all the cases. In this research, it is shown that the blood flow pattern in MI LV is significantly different from that in the normal LV. For instance, as shown in Fig. 5, the number and strength of the main vortices of normal LV models are larger and stronger than MI models at the peak of systole. Also, more small vortices are generated in a normal LV at end-diastole, as shown in Fig. 5. The results show that the flow momentum in MI models is lower than in the normal models due to the enlarged volume. Contrary to normal cases, the pressure difference (and pressure gradient) is considerably lower in the patient models due to the low stroke volume. Based on these obtained results, the researchers observed that a quantitative assessment of the blood flow pattern and vortices could assist the early diagnosis of heart dysfunction.

**Fig. 6:**
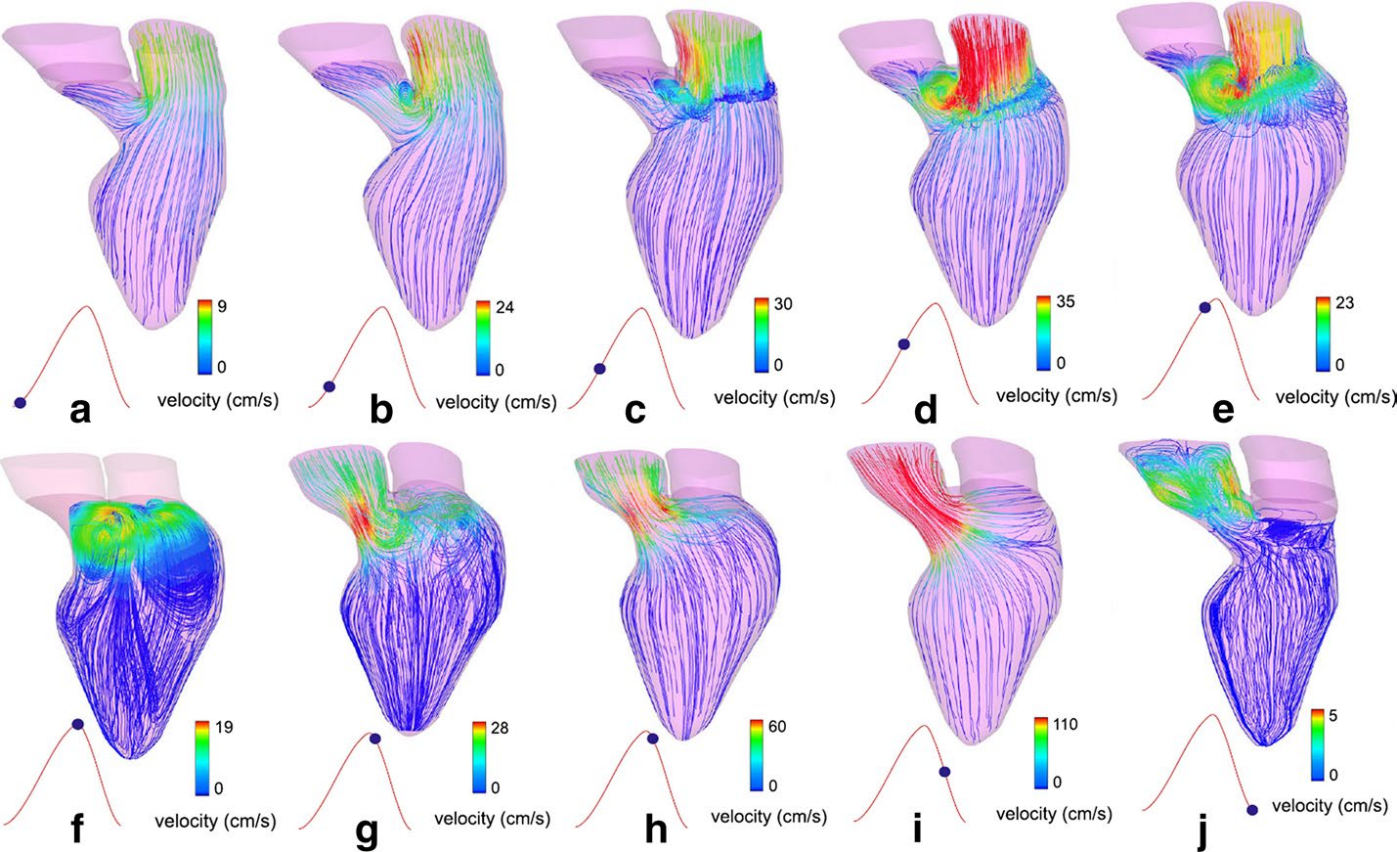
Flow patterns of an MI patient before surgery: The flow patterns are shown during diastole (**a**–**f**) and during systole (**g**–**j**) respectively. Vortices during diastole disappear quickly after their formation in the preoperative case [13] (Adapted from [13], with permission from Wiley)

**Fig. 7:**
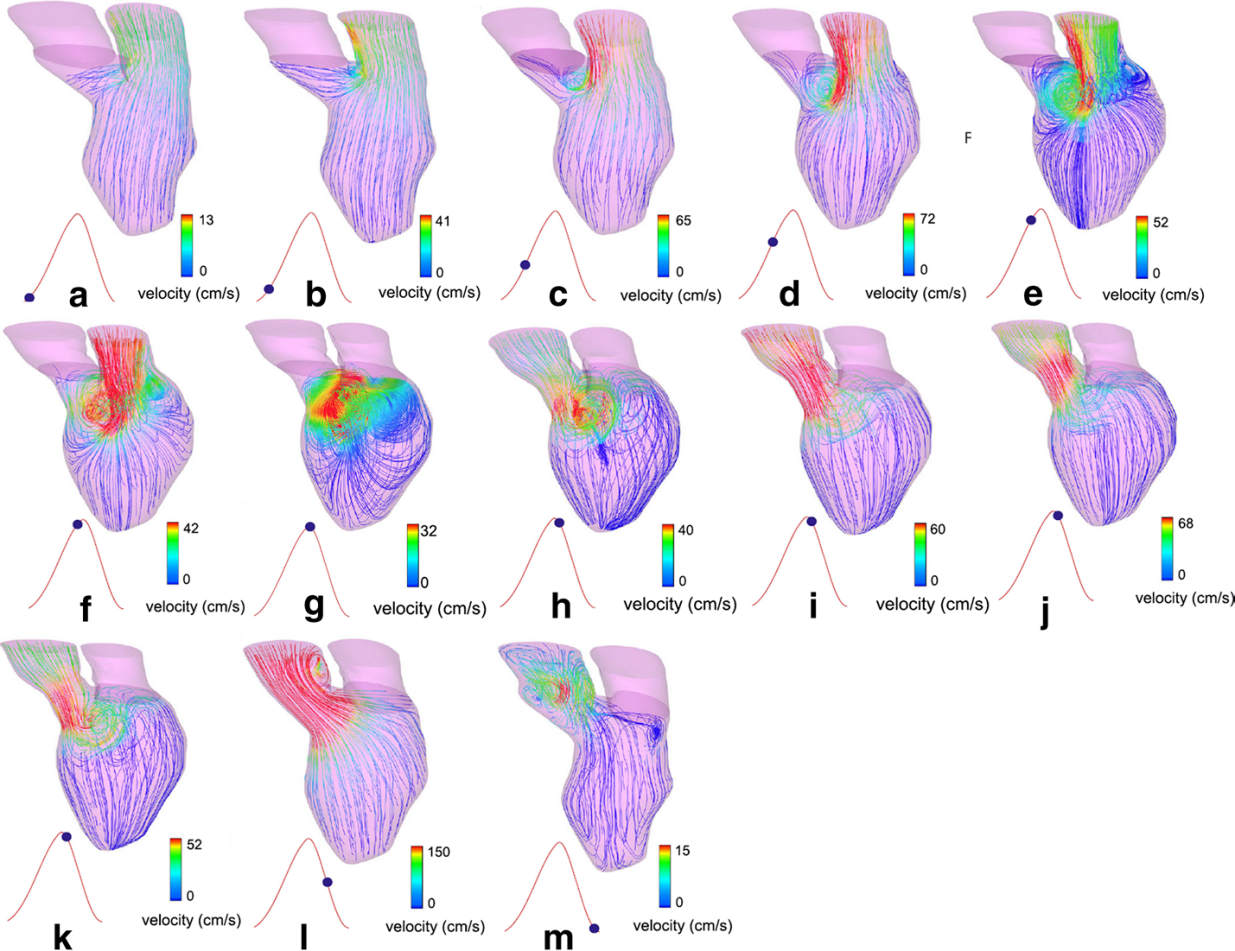
Flow patterns of an MI patient after surgery: Flow pattern during diastole (**a**–**f**) and systole (**g**–**m**), respectively. Strong vortices are formed during diastole in comparison to the pre-operative model (Fig. 6), which demonstrates the improvement in blood flow circulation after SVR. Improvement of the outflow jet direction through the aortic orifice demonstrates more efficient blood pumping after operation [13] (Adapted from [13], with permission from Wiley)

Subsequently, Khalafvand et al. [31] compared the hemodynamic parameters of one patient LV before and 4 months after SVR surgery, to observe the surgery outcome from a hemodynamic point of view. In this research, unlike in their previous study, they used 3D models of the preoperative and postoperative LV to compute the blood flow dynamics. They illustrated that SVR surgery enhanced the strength of the intraventricular vortices that led to a higher ejection fraction during the cardiac cycle. Later, they [13] further investigated the influence of the SVR and coronary artery bypass grafting (CABG) surgery in the patient-specific model before and after the surgery. The flow patterns in both the LV models before and after the SVR are shown in Figs. 6 and 7. The results show that the vortices in the preoperative model are weak in comparison to the postoperative model. The results also show that the maximum velocities at the inlet and outlet orifices in the preoperative model are less than postoperatively. The results demonstrate that during diastole, stronger vortices are generated in the postoperative model, which improves blood recirculation. Vortices are noted to disappear quickly after their formation in the preoperative case, but stay longer in the postoperative model. In both cases, the direction of the main vortex enables efficient ejection during the systolic phase. Likewise, the ejection fraction shows improvement from 34 to 48 % after SVR. These results demonstrate the effectiveness of SVR to improve intraventricular flow patterns and produce (i) stronger vortices during the cardiac cycle, and (ii) a higher ejection fraction. Therefore, these results illustrate that CFD can be utilized to investigate surgery outcomes.

Likewise, Doenst et al. [35] numerically studied the intraventricular hemodynamics of preoperative and postoperative patient-specific LVs, to investigate the effectiveness of SVR surgery on the remodeled LV. The result shows that the postoperative LV geometry is more spherical in comparison to the preoperative LV and normal LV. The intraventricular flow pattern after SVR is significantly different from the flow pattern before surgery, but is still not as good as that of the healthy LV. The flow patterns after surgery and in the normal LV are topologically similar during the diastolic phase. The streamlines before surgery show a stagnation point in the apex region; also, the vortices are not expanding asymmetrically inside the LV cavity, which prevents blood flow redirection toward the aortic outflow track. The numerical results demonstrate that the washout volume of the normal LV after four cardiac cycles is 2 %, but the value for the preoperative LV is 35 % and for the postoperative LV is just slightly less than 35 %. This shows that the LV washout after surgery is not considerably improved in comparison to the preoperative LV in spite of the large shape modification. The ejection fractions in the normal, preoperative and postoperative LV are 0.61, 0.15, and 0.18, respectively. Therefore, the intraventricular hemodynamics improvement contributes to the enhanced postoperative ejection fraction.

#### Dilated cardiomyopathy (DCM)

Dilated cardiomyopathy (DCM) is another pathological heart condition causing ventricular dilatation and heart enlargement. The DCM condition progressively reduces the contractility of the LV by changing the natural heart shape and size. This pathological condition reduces the development of adequate systolic pressure due to decreased LV contractility, and thereby leads to reduced cardiac output [63]. As in the MI condition, the heart’s hemodynamic parameters change in the DCM condition due to heart remodeling. In the DCM condition, the intraventricular vortices become weaker and smaller due to flow momentum reduction in the enlarged LV. Hence, CFD simulation by patient-specific models and comparison with healthy LV models, and finding the correlation between the hemodynamic parameters and the ventricular performance can enhance our knowledge about the progress and severity of DCM.

To characterize intraventricular flows in DCM patients, Mangual et al. [7] numerically and statistically analyzed the hemodynamic parameters of 20 normal subjects and 8 DCM patients by using a combination of 3D echocardiography and Direct Numerical Simulation methods. Statistical results show that the ejection fraction in DCM patients (17.8 ± 6.4 %) is significantly lower than in a normal heart (55.4 ± 3.5 %). The numerical finding indicates that, during mid-diastole, a counter-clockwise vortex is developed in the entire LV cavity for the normal subject; however, for the DCM patient, a small vortex ring is generated on the upper side of the LV cavity. Moreover, at end-diastole, the large vortex ring in the normal subject is redirected to the outflow track; in the DCM patient, a weak vortex is formed and is located in the middle of the LV cavity. The results also show that the vortex formation time in the normal LV is considerably greater than in the case of the DCM patient. Moreover, the kinematic energy dissipation in the normal LV during diastole and systole is more than in the normal LV.

#### Hypertrophic cardiomyopathy (HCM)

**Fig. 8:**
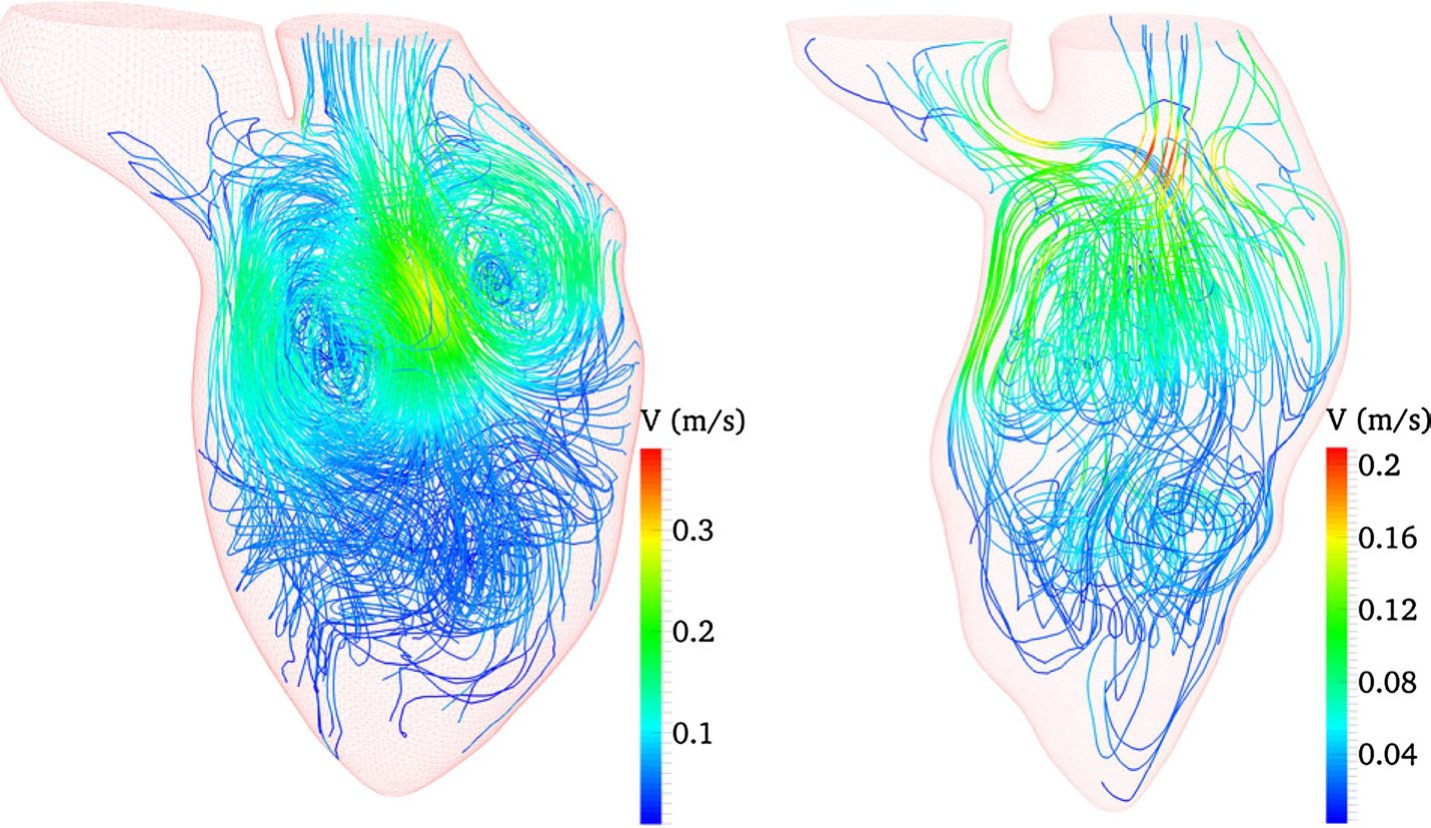
Comparison of intraventricular flow patterns in a normal subject and a HCM patient: Intraventricular streamline distributions at the end of diastole in a healthy subject model (*left*) and a HCM patient model (*right*). It is seen that larger and stronger vortices are developed in the healthy LV. Also, the vortices are pumped deeply into the apex part in the HCM LV [22] (Reprinted from [22], with permission from IEEE)

**Fig. 9:**
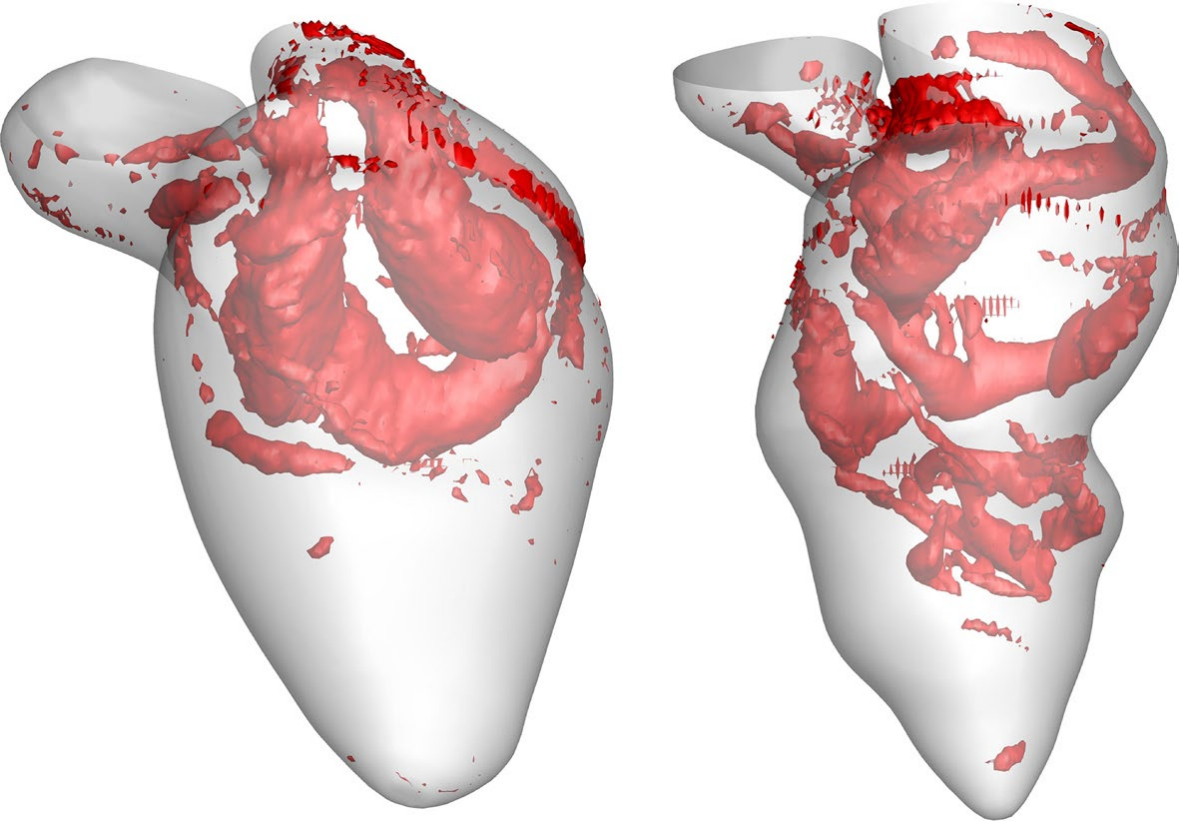
Comparison of end-diastolic vortex formation in a normal subject and a DCM patient. The vortex structures of one healthy (*left*) and HCM (*right*) model are compared. The major vortex structure remains strong, like a cirrostratus cloud, at the end of diastole. The major vortex in the disease model is rolled up deeply toward the apex, and it is dissipated into connected small vortices [22] (Reprinted from [22], with permission from IEEE)

Hypertrophic cardiomyopathy (HCM) is a myocardial defect that refers to an excessive thickening of a portion of the LV myocardium that causes sudden HF. The HCM condition and the resulting LV stiffness interferes with the ability of the LV to expand and fill before the onset of systole, due to the LV size and myocardium elasticity reduction [64]. The myocardium thickening and the flow obstruction in the HCM pathological condition have a strong impact on LV performance and the intraventricular blood flow. Therefore, the CFD simulation of the HCM LV can provide useful insights for understanding the variation of the intraventricular blood flow dynamics in this disease condition. To study the effect of HCM, Su et al. [22] simulated the flows in a normal subject and a HCM LV, in order to compare the intraventricular flow patterns of the HCM LV and healthy LVs. In this study, they thoroughly compared the formation and propagation of the intraventricular vortices in different cardiac stages. As shown in Fig. 8, larger and stronger vortices are developed in the healthy LV in comparison to the HCM LV at the end of diastole. Also, the vortex ring growth is disrupted in the HCM LV in comparison with the healthy LV due to the narrowing of the LV chamber. As seen in Fig. 8, vortices are pumped deeply into the apex part in the HCM LV. Moreover, as shown in Fig. 9, a comparison of the vortex structures in the two models shows that a cirrostratus-like cloud is formed in the HCM LV, while a normal major vortex ring is formed in the healthy LV.

#### Hypoplastic left heart syndrome (HLHS)

**Fig. 10:**
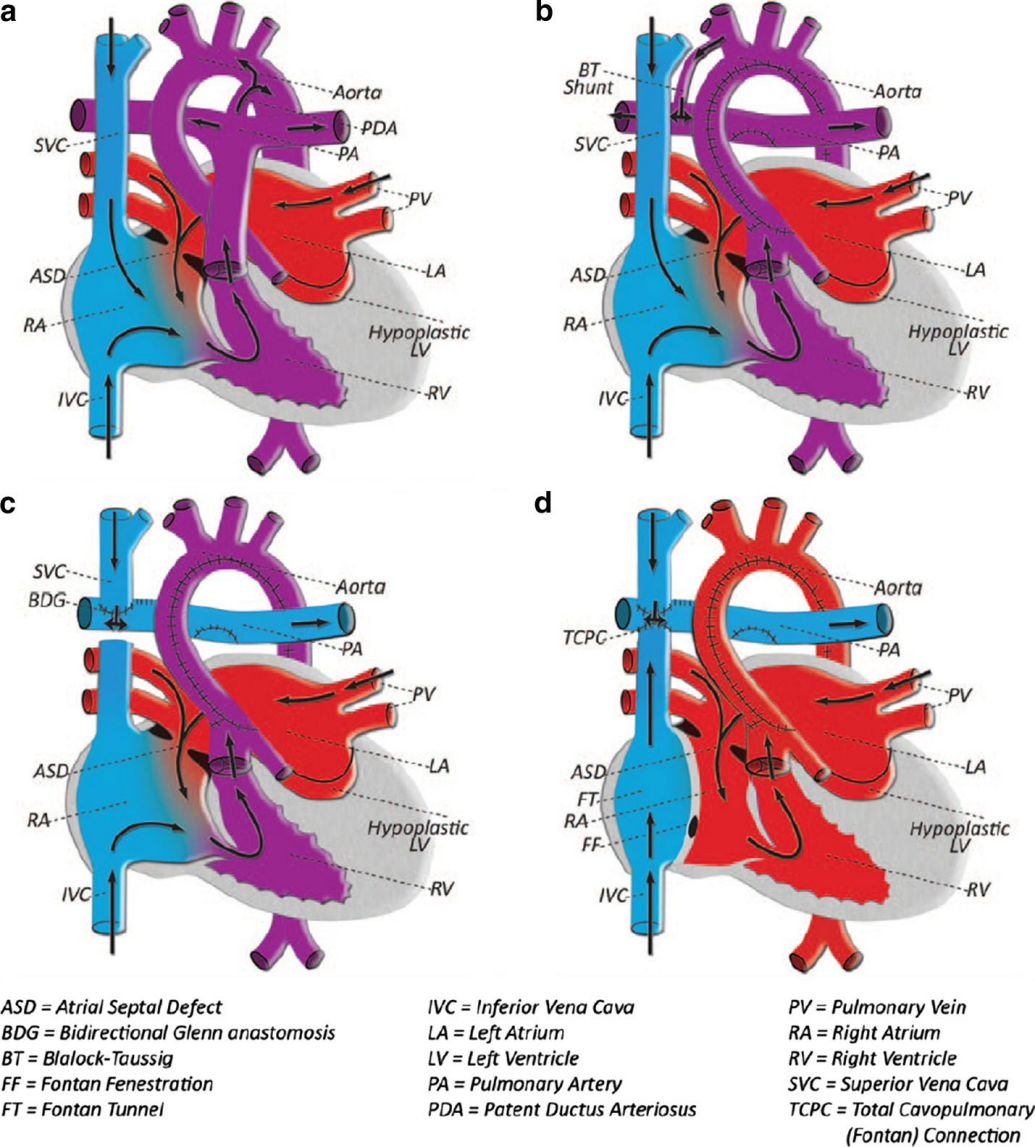
Different stages of operations performed on patients with HLHS: **a** The general schematic of the heart in the HLHS condition; RV supports both pulmonary and systemic circulations. **b** Stage I (Norwood): the ascending aorta and aortic arch is reconstructed, and a shunt is inserted between the pulmonary artery and subclavian vessel, **c** Stage II (Glenn): the superior vena cava is connected to the pulmonary artery to isolate the pulmonary circulation, **d** Stage III (Fontan): both superior and inferior vena cave arteries are connected to the pulmonary to completely isolate the pulmonary and systemic circulations [67] (Reprinted from [67], with permission from Macmillan Publishers Ltd)

The hypoplastic left heart syndrome (HLHS) is a congenital heart disorder that refers to an underdeveloped LV before birth. In the HLHS condition, the RV supports both pulmonary and systemic circulations. This heart defect is a fatal condition that needs surgery in the first days after birth. As shown in Fig. 10, complex multistage surgery must be performed to isolate the pulmonary and systemic blood circulations. Usually, there are three stages in the operation, these being Norwood, Glenn, and Fontan [65]. In the first stage of the operation, the Norwood operation, the ascending aorta and aortic arch is reconstructed by using the pulmonary artery to create systemic circulation. Subsequently, a shunt is inserted between the pulmonary artery and subclavian vessel in order to maintain pulmonary circulation. In the second stage, the Glenn operation, the pulmonary circulation is isolated from the systemic circulation by connecting the superior vena cava to the pulmonary artery. However, the deoxygenated blood received from the inferior vena cava still mixes with the oxygenated blood in systemic circulation. Finally, both superior and inferior vena cave arteries are connected to the pulmonary artery in the third stage, the Fontan operation, in order to completely isolate the pulmonary and systemic circulations. At the end of the third operation, the RV pumps only oxygenated blood to the systemic circulation [26, 66, 67]. This multistage operation is complex and has high risk; therefore, numerical simulations of each stage prior to surgery can be a useful and promising tool. Some numerical investigations [65, 68] have been carried out to evaluate the ventricular workload of the single ventricle by using different types of arch reconstruction and calculating the hemodynamic factors, such as energy loss and WSS. For instance, the numerical findings of utilizing various Norwood arch reconstruction in [68] suggested that using a smooth aortic arch angle with the large anastomotic space lead to the reduction of WSS and energy loss, meaning the improvement of postoperative cardiac performance.

In order to investigate the effect of aortic arch reconstruction on the functionality of the postoperative RV, Vecchi et al. [26] numerically studied intraventricular blood flows in two different patient-specific HLHS cases after aortic arch reconstruction and compared them with the flow in the normal LV. The numerical findings show that the filling streamlines and the myocardial displacements of the two HLHS RV cases and a healthy normal LV are significantly different at the peak of E-wave. The numerical results demonstrate that the shape and propagation of the vortex are completely different in the two HLHS cases in comparison with the normal case. The high velocity difference between the basal and apical region reduces the diastolic process efficiency due to the reduced pressure gradient. Thereby, it can be seen that the reduced and/or delayed early pressure gradient is associated with LV diastolic dysfunction. In 2013, Corsini et al. [16] numerically simulated preoperative and postoperative patient-specific models to study the outcome of the stage two single ventricle (SV) surgery. The 3D virtual surgery was performed with two different surgical options (hemi-Fontan operation and bi-directional Glenn) in the preoperative model, to investigate the performance of both surgeries from a hemodynamic point of view. Even though the numerical post-operative results show little difference in the local hemodynamics between the two surgery options, the study shows the capability of CFD in selecting the optimal surgical option before the operation.

## Validation of numerical findings

Verification can be defined as “solving the equations right”, which in turn assesses the accuracy of the numerical data by using analytical solutions. Computational method validation on the other hand can be defined as “solving the right equations”, and validating the numerical predictions with real or experimental data [69]. The validity of the cardiovascular CFD simulation results widely depends on the selection of appropriate geometry, boundary conditions, fluid and solid domain material property, mesh qualities, and the numerical approach. Due to the many simplifications and assumptions taken into account in the numerical simulation of LV, the degree of accuracy of the results needs to be assessed prior to utilizing them for applications in clinical practice. However, because of the difficulty in measuring the hemodynamics parameters of the cardiovascular system, only a few papers have validated their numerical findings. In some publications, such as [8], only a qualitative validation is available by utilizing in vivo magnetic resonance velocity imaging. A quantitative comparison of CFD results and magnetic resonance measurements in LV simulation is challenging in comparison with flow simulation in large arteries, due to the complex nature of the intraventricular flow pattern and large deformation of the LV geometry [8]. Also, a circulatory system with a pressurized chamber to reproduce physiological flow, similar to the LV, has been used in [34, 35] to qualitatively validate the numerical findings of the intraventricular flow dynamics.

Saber et al. [39] have quantitatively compared the intraventricular blood flow patterns obtained by CFD simulation with the in vivo measured data in previous work [70, 71] obtained by magnetic resonance velocity mapping. Long et al. [8] have qualitatively validated their numerical simulation results, using a similar technique. The MRI images detected small vortices close to the inflow tract and papillary muscles, which were not observed in the CFD simulation due to geometry simplifications. Another qualitative validation of numerical results using in vivo flux mapping was performed by Schenkel et al. [36]; in vivo flux mapping was performed by using the MRI phase coded flux scan with 3-directional flow velocity encoding. Overall, the velocity contours extracted from CFD simulation were found to be in good agreement with the MRI flux measurements.

Krittian et al. [34] developed an artificial ventricular setup to validate the numerical simulation of the LV, which was performed by using two different approaches: (1) geometry-prescribed (KaHMo MRT), and (2) the coupled-FSI (KaHMo FSI). The experimental setup consists of a simplified LV sac that is integrated with biological heart valves. The LV sac was placed in a pressurized chamber to reproduce physiological flow, and the flow pattern was captured by using the Particle Image Velocimetry (PIV) technique. In this study, it has been shown that the blood flow pattern was in good qualitative agreement with the experimental results. The experimental results represented the capability of numerical simulation to reproduce an approximately similar flow pattern formed in the experimental setup. Moreover, the numerical and experimental results show that other hemodynamic and structural parameters, such as the LV cavity spatiotemporal structural volume deformation, LV pump characteristics (such as the pressure–volume work, performance, mixing coefficients, and ejection fraction) and the cardiac cyclic pressure–volume relationship are in a good agreement.

## Conclusion

In this review paper, we have presented the various investigations that have been conducted to numerically simulate patient-specific human LVs over the past 15 years by using IB-CFD methods.

### CFD hemodynamic parameters utilization for detailed characterization

CFD is considered to be a robust tool that can be used to evaluate the hemodynamic parameters of intraventricular blood flow, such as WSS, pressure distribution, pressure gradient or other intraventricular blood flow parameters, to facilitate the detailed characterization of LV pathologies. The recent advancement of blood flow modeling can provide a detailed understanding of the blood flow dynamics, which cannot be achieved solely through invasive modalities, such as characterization, or medical imaging. The computer modeling of the intraventricular flow fulfills the capability of hemodynamic parameters to serve as non-invasive clinical diagnostic indices, to facilitate diagnosis of LV dysfunction [72]. Vascular hemodynamics, involving numerical simulation of blood flow in arteries, is now widely accepted for use in clinical practices. Now it is a welcome news that HeartFlow^®^ FFR_CT_ software (HeartFlow Inc., USA) has received the FDA approval for clinical applications [73]; however, we still need to take care of the heart flow simulation challenges, such as incorporating heart valve motion. In the meantime, we can be in the process of deciding which hemodynamic parameters can be best utilized to assist physicians in the early diagnosis and prognosis of CVDs.

### Benefits of IB-CFD patient-specific intraventricular flow modeling

Patient-specific LV models can be used for various purposes, such as for (i) hemodynamic evaluation of physiological and pathological LVs, and (ii) assessment of surgery outcomes by analyzing preoperative LVs and simulating the hemodynamics associated with the various surgical alternatives prior to performing surgery, i.e. the virtual surgical planning. Objectively speaking, IB-CFD patient-specific intraventricular flow modeling has the potential to become a viable tool for: (i) assessing LV pathologies for clinical practice, and (ii) determining how reconstructive surgical procedures can improve cardiac functional performance.

This study has notably revealed that different targets have been selected by authors to numerically simulate the LV flow dynamics, such as (i) characteristics analysis [2], (ii) analysis of preoperative and postoperative LVs to evaluate surgical outcomes [13], (iii) preoperative LV analysis to examine various surgical alternatives to choose the best option [16], and finally (iv) analysis of pathological LVs to assess their physiological conditions [17].

### Some concerns in relation to IB-CFD patient-specific modeling

For the purpose of further improvements in diagnostics, prognosis and surgical outcomes, it is worthwhile mentioning some limitations of and concerns in relation to IB-CFD patient-specific LV modeling and analysis. The IB-CFD requires high operator-dependent steps, such as image acquisition, image segmentation, geometry reconstruction, mesh generation, and finally numerical simulation [27]; these steps can be potential sources of error that can impact the results. In addition, other CFD errors can arise, such as the round-off error, iterative error, convergence error, as well as the possibility of defining inappropriate boundary conditions. Moreover, the numerical instability and the convergence criteria of the CFD problem are other concerns relating to numerical simulations. Additionally, an LV CFD simulation study usually needs parallel processing and more computing facilities, which makes it somewhat expensive and time-consuming. Also, most of the available models include some geometrical and/or physical approximations/assumptions that can affect the computational results.

### Further improvements in LV CFD simulation

A more precise model to mimic realistic hemodynamics of patient-specific LVs needs to include the following elements:

1. More realistic geometry, including the physiological inner endocardium surface, papillary muscles, and chordae tendineae,
2. Simulation of the actual heart mitral and aortic valves motion,
3. Incorporation of realistic blood properties (non-Newtonian properties) and myocardium structural properties,
4. EFSI of the LV,
5. Reconstruction of other associated cardiovascular components, such as the LA, aortic root, and valves in order to provide a more realistic boundary condition.

### LV CFD Simulation could constitute a promising clinical tool, with the inclusion of the following several improvements in the future researches

(i) data acquisition techniques to capture high spatiotemporal resolution images, (ii) image processing techniques to reconstruct precise geometry, (iii) computing facilities to simulate the model in a short time period, and (iv) more rigorous correlation of the hemodynamic parameters with the clinical quantification of heart dysfunctional assessment and its improvement by surgical procedures. Finally, as stated in [74], a multidisciplinary collaboration between clinicians and engineers is required to understand the approximations, assumptions, and limitations of the numerical simulations in order to utilize CFD findings in clinical decisions.

Altogether, we can say that heart flow simulation is on the right track for developing into a useful clinical tool for heart function diagnosis. Heart flow simulation now needs to determine some diagnostic indices based hemodynamic parameters, which we can start adopting in clinical usage. In the meantime, we also need to work on incorporating most of heart structures’ (such as heart valves) operations into our heart hemodynamics modeling, so as to most closely simulate intraventricular flow.

## Abbreviations

BMHV: bileaflet mechanical heart valve
CABG: coronary artery bypass grafting
CFD: computational fluid dynamics
CT: computed tomography
CVD: cardiovascular disease
DCM: dilated cardiomyopathy
ECG: echocardiography
EFSI: electrical-fluid-structure interaction
FSI: fluid-structure interaction
HF: heart failure
HCM: hypertrophic cardiomyopathy
HLHS: hypoplastic left heart syndrome
IB-CFD: imaged-base computational fluid dynamics
IBM: immersed boundary method
LA: left atrium
LES: large eddy simulation
LHF: left heart failure
LV: left ventricle
MI: myocardial infarction
MRI: magnetic resonance image
PIV: particle image velocimetry
PAH: pulmonary arterial hypertension
RV: right ventricle
SV: single ventricle
SVR: surgical ventricular reconstruction
WSS: wall shear stress
